# Progressive Reduction of its Expression in Rods Reveals Two Pools of Arrestin-1 in the Outer Segment with Different Roles in Photoresponse Recovery

**DOI:** 10.1371/journal.pone.0022797

**Published:** 2011-07-26

**Authors:** Whitney M. Cleghorn, Elviche L. Tsakem, Xiufeng Song, Sergey A. Vishnivetskiy, Jungwon Seo, Jeannie Chen, Eugenia V. Gurevich, Vsevolod V. Gurevich

**Affiliations:** 1 Department of Pharmacology, Vanderbilt University, Nashville, Tennessee, United States of America; 2 Department of Cell and Neurobiology, University of Southern California, Los Angeles, California, United States of America; Universidade Federal do Rio de Janeiro, Brazil

## Abstract

Light-induced rhodopsin signaling is turned off with sub-second kinetics by rhodopsin phosphorylation followed by arrestin-1 binding. To test the availability of the arrestin-1 pool in dark-adapted outer segment (OS) for rhodopsin shutoff, we measured photoresponse recovery rates of mice with arrestin-1 content in the OS of 2.5%, 5%, 60%, and 100% of wild type (WT) level by two-flash ERG with the first (desensitizing) flash at 160, 400, 1000, and 2500 photons/rod. The time of half recovery (t_half_) in WT retinas increases with the intensity of the initial flash, becoming ∼2.5-fold longer upon activation of 2500 than after 160 rhodopsins/rod. Mice with 60% and even 5% of WT arrestin-1 level recovered at WT rates. In contrast, the mice with 2.5% of WT arrestin-1 had a dramatically slower recovery than the other three lines, with the t_half_ increasing ∼28 fold between 160 and 2500 rhodopsins/rod. Even after the dimmest flash, the rate of recovery of rods with 2.5% of normal arrestin-1 was two times slower than in other lines, indicating that arrestin-1 level in the OS between 100% and 5% of WT is sufficient for rapid recovery, whereas with lower arrestin-1 the rate of recovery dramatically decreases with increased light intensity. Thus, the OS has two distinct pools of arrestin-1: cytoplasmic and a separate pool comprising ∼2.5% that is not immediately available for rhodopsin quenching. The observed delay suggests that this pool is localized at the periphery, so that its diffusion across the OS rate-limits the recovery. The line with very low arrestin-1 expression is the first where rhodopsin inactivation was made rate-limiting by arrestin manipulation.

## Introduction

Humans express ∼800 different G-protein-coupled receptors (GPCR), among which rhodopsin is the best characterized [1]. The biochemical mechanism of rod phototransduction serves as a model of GPCR-driven signaling cascades [1]. Rhodopsin is activated by photoconversion of covalently attached retinal. Light-activated rhodopsin catalyzes nucleotide exchange of cognate G protein transducin, which then activates cGMP phosphodiesterase. Rhodopsin is inactivated by GRK1 phosphorylation, followed by high-affinity binding of arrestin-1 when three attached phosphates are accumulated [2], [3]. Comprehensive understanding of systems behavior of rod photoreceptors requires the knowledge of exact concentration, localization, and activity of every signaling protein in the cell. While the functional role of many players in rod phototransduction have been qualitatively established using genetically modified mice (reviewed in [4]), the biological significance of the exact expression level of each protein was rarely addressed experimentally. The studies where rods with different expression levels of rhodopsin [5], [6], RGS9 [7], [8], GRK1 [9], and arrestin [8]–[10] were functionally characterized yielded important, often surprising, results. Mouse rods express arrestin-1 and rhodopsin at ∼0.8∶1 ratio, which makes arrestin-1 the second most abundant protein in the rod photoreceptor [10]–[12]. Using transgenic mice expressing arrestin-1 at levels ranging from 4 to 220% of WT, we recently found that supra-physiological arrestin-1 levels marginally improve the functional performance of rods [10]. In addition, rod photoreceptors with arrestin-1 levels below WT perform as well as other genotypes at dim light, but show dramatic functional impairment when tested at brighter illumination. [10]. Importantly, the reduction of arrestin-1 level in the OS to ∼2.5% of WT dramatically slowed the recovery kinetics, as compared to mice with only twice as much arrestin-1 in the OS [10]. Here we show that, while the recovery rates in all lines slow with the increased intensity of the desensitizing flash, the same “threshold” between 5% and 2.5% of arrestin-1 level in the OS is observed at all flash intensities tested. Remarkably, this threshold is maintained even at the dimmest desensitizing flash. These data indicate that ∼2.5% of arrestin-1 content in the OS is not immediately available for rhodopsin quenching, suggesting that this separate pool of arrestin-1 resides relatively far from rhodopsin-containing discs. Slow diffusion of arrestin-1 across the OS in the lowest expressing line apparently delays the recovery by making rhodopsin inactivation rate-limiting, in contrast to WT and arrestin-1 hemizygous (Arr1+/−) animals where transducin inactivation is the slowest process that determines the speed of recovery [7], [8], [13]. Please note that we use systematic names of arrestin proteins: arrestin-1 (historic names S-antigen, 48 kDa protein, visual or rod arrestin), arrestin-2 (β-arrestin or β-arrestin1), arrestin-3 (β-arrestin2 or hTHY-ARRX), and arrestin-4 (cone or X-arrestin; for unclear reasons its gene is called “*arrestin 3*” in HUGO database).

## Results

**Figure 1:**
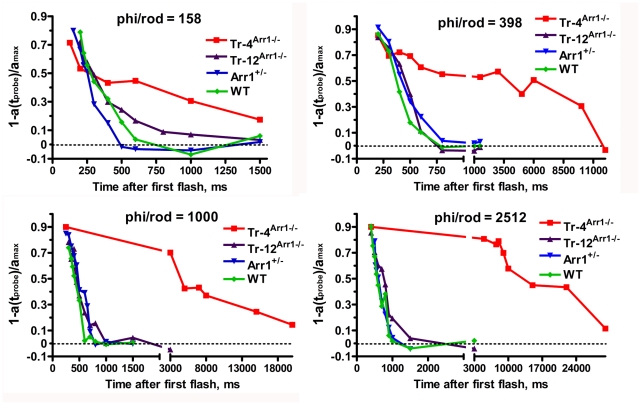
Reduced arrestin-1 expression slows down photoresponse recovery. The intensities of the first (desensitizing) flashes were −0.8, −0.4, 0, or +0.4 *log*cd*s/m^2^ and second (probe) flash was 0.65 *log*cd*s/m^2^. The a-wave elicited by the probe flash was plotted as a function of time elapsed after the first flash. Representative recovery curves for indicated genotypes and strengths of desensitizing flash are shown. The interval between the two flashes was varied from 200 to 120,000 ms. The results for desensitizing flash of −0.4 *log*cd*s/m^2^ were reported previously [10], and are shown here for comparison. Phi/rod, photoisomerizations/rod. [59]

**Figure 2:**
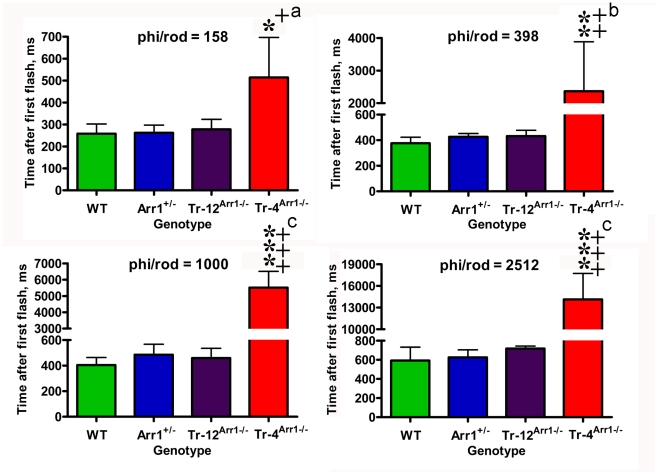
Animals with very low arrestin-1 in the OS show very long time of half recovery. To calculate the time of half recovery, recovery kinetics were fitted by polynomial nonlinear regression, with R^2^>0.95, as described in methods. Means +/− SD for four animals per genotype are shown. The data were analyzed by one-way ANOVA with Genotype as main factor followed by Bonferroni post hoc comparison of means. * - p<0.05; ** - p<.001, *** - p<0.001 to WT; + - p<0.05, ++ - p<0.001, +++ - p<0.001 to Arr+/−, a – p<0.005, b – p<0.01, c – p<0.001 to Tr-12^Arr−/−^. Phi/rod, photoisomerizations/rod.

Arrestin-1 binding after GRK1 phosphorylation of rhodopsin [14], [15] is the key process in rapid photoresponse recovery in rods [16] and cones [17]. Arrestin-1 acts by sterically shielding rhodopsin, precluding further transducsin activation [18], [19]. In the dark, arrestin1 translocates out of OS and localizes primarily to cell bodies of rod photoreceptors, so the OS contains only a small proportion of arrestin-1 [10]–[12], [20]–[22]. Dark-adapted rod OS of transgenic mice expressing arrestin-1 at 4% (Tr-4^Arr−/−^), 12% (Tr-12^Arr−/−^), 50% (Arr+/−), and 100% of WT contain ∼7.6, 15, 180, and 300 µM arrestin-1, respectively (these calculations are based on 3 mM rhodopsin concentration in the OS [10], [23]), which constitutes 2.5%, 5%, 60%, and 100% of normal WT level, respectively. Rod function can be monitored non-invasively by ERG, where the negative a-wave reflects the hyperpolarization of the rod cells caused by reduction of circulating current [24]–[27]. We used double-flash protocol, where an initial flash desensitizes rods, and the response to the second (probe) flash, delivered at varying time intervals after the initial flash, is measured to determine the extent of recovery. The time of half-recovery (t_half_) is calculated by plotting the amplitude of the probe flash response as a function of time between flashes [24], [28]. Using desensitizing flash of −0.4 *log*cd*s/m^2^ (400 photoisomerizations/rod), we previously found that recovery rates of the three lines with 100%, 60%, and 5% of WT arrestin-1 level in the OS are surprisingly similar, whereas rod recovery in mice with 2.5% of normal arrestin-1 content in the OS is dramatically slowed (Fig. 1) [10]. Considering that the pseudo-first-order rate of arrestin-1 binding to phosphorylated rhodopsin is the product of the on-rate constant (which was recently measured [29]) multiplied by the absolute arrestin-1 concentration near rhodopsin-containing discs, two mechanistic models could account for this “threshold”-like effect. If arrestin-1 is homogeneously distributed throughout OS cytoplasm, the threshold must depend on the intensity of the desensitizing flash, so that the activation of more than twice as many rhodopsins should place Tr-12^Arr−/−^ mice with two-fold greater arrestin-1 content below the threshold. Alternatively, arrestin-1 distribution in the OS may be non-homogeneous, with immediately available and relatively unavailable pools. If the latter pool is roughly equal to arrestin-1 content in the lowest expressing animals, Tr-4^Arr−/−^ mice would be below the threshold at all intensities of desensitizing flash, whereas all other lines would remain above it. To distinguish between these two possibilities, we used initial desensitizing flashes with intensities that vary ∼16-fold, −0.8, −0.4, 0, and +0.4 *log*cd*s/m^2^, corresponding to 160, 400, 1000, and 2500 photoisomerizations/rod (Figs. 1,2; Table 1) [30]. Unexpectedly, we found no significant differences in the t_half_ of WT, Arr+/−, and Tr-12^Arr−/−^ mice at any intensity of desensitizing flash tested, despite ∼20-fold difference in the arrestin-1 content in the OS of WT and Tr-12^Arr−/−^ animals. However, the magnitude of the recovery defect in Tr-4^Arr−/−^ mice depended on flash intensity. At 160 photoisomerizations/rod, t_half_ of Tr-4^Arr−/−^ mice was only ∼1.8-fold longer than in other genotypes, but the difference increased to ∼5.5-, 12-, and 23-fold at 400, 1000, and 2500 photoisomerizations/rod, respectively (Fig. 2; Table 1).

**Table 1:** The rates of photoresponse recovery in mice with different arrestin-1 expression.

Genotype	158 phi/rod	398 phi/rod	1000 phi/rod	2512 phi/rod	Arrestin-1 concentration (OS)
Wild type	258±45 ms	376±47 ms	405±58 ms	646±56 ms	300 µM
Arr1^+/−^	262±35 ms	426±26 ms	486±82 ms	626±79 ms	180 µM
Tr-12^Arr1−/−^	278±46 ms	433±45 ms	460±75 ms	718±27 ms	15 µM
Tr-4 ^Arr1−/−^	514±183 ms	2368±1515 ms	5515±999 ms	14137±3595 ms	7.6 µM

Importantly, WT, Arr+/−, and Tr-12^Arr−/−^ mice demonstrated a gradual slowing of the recovery with increasing intensity of the desensitizing flash, and the slope of the slowing was the same in these three genotypes, as evidenced by lack of interaction between Genotype and Light factors in two-way-ANOVA (F(6,41)-1.12, p = 0.37 n.s.). In these genotypes t_half_ increased ∼2.5-fold with desensitizing flash inducing 2,500 instead of 160 photoisomerizations/rod (Table 1). In sharp contrast, the increase in recovery time from the dimmest to brightest desensitizing flash for Tr-4^Arr−/−^ mice was ∼28 fold (Table 1). Virtually identical slowing of the recovery in WT, Arr+/−, and Tr-12^Arr−/−^ animals likely reflects the increased time that it takes guanylyl cyclase to replenish hydrolyzed cGMP necessary to open cGMP-gated channels and restore circulating current, whereas much more dramatic increase of t_half_ in Tr-4^Arr−/−^ animals must reflect additional processes that do not operate in the other three genotypes.

## Discussion

Rod phototransduction is the only GPCR-driven signaling cascade where the expression levels of all players are known with sufficient accuracy to model systems behavior of the cell [13], [31]–[33] Here we report an unexpected finding that 20-fold reduction of arrestin-1 content in the dark-adapted rod OS from 100% to 5% of WT level has no appreciable effect on photoresponse recovery, whereas further 2-fold reduction to 2.5% dramatically slows this process (Figs. 1,2; Table 1). This remarkable difference in recovery kinetics is unlikely to be simply the result of depletion of arrestin-1 in the OS. There are ∼70 million rhodopsin molecules in the mouse OS [30], clustered on approximately 800 discs. This corresponds to ∼88,000 rhodopsins per disc. The calculated amount of arrestin-1 present in the OS for Tr-4^Arr−/−^ mice is approximately 7.6 µM, which corresponds to about 200,000 molecules per OS [10]. In the case of even arrestin-1 distribution in the OS, there would be ∼250 arrestin-1 molecules per disc available to quench rhodopsin. However, even at the dimmest desensitizing flash used, which generates only 160 Rh*/rod (0.2 Rh*/disc), we observed a 1.8-fold slowing of the recovery, which increases to >20-fold at ∼3 Rh*/disc (Table 1). Arrestin-1 concentration in the WT mouse OS is ∼300 µM [10]. Taking into account known constants of mouse arrestin-1 self-association [34], this yields ∼50 µM active monomer. This results in estimated pseudo-first-order on-rate of 50 s^−1^, enabling arrestin-1 to “check” the state of each rhodopsin molecule every 20 msec. This is consistent with recent estimates of an active rhodopsin lifetime of <60 ms [7], or possibly even ∼30 ms [8], [13]. Calculations show that despite the dramatically reduced arrestin-1 concentration in Tr-4^Arr−/−^ mice, at 7.6 µM there is still enough arrestin-1 monomers in the OS to encounter each rhodopsin every 200 ms. This difference is sufficient to account for ∼200 ms delay, but cannot explain the multi-second times of half-recovery observed (Fig. 2; Table 1). Thus, our data suggest that most of arrestin-1 in the OS of Tr-4^Arr−/−^ animals is not immediately available for rhodopsin quenching.

Self-association could potentially limit arrestin-1 availability. Arrestin-1 forms dimers and tetramers at physiological concentrations [35]–[37], yet only the monomer is capable of binding rhodopsin [37], [38], because the well-defined rhodopsin-binding surface of each molecule [39]–[47] is occluded by other subunits in the solution tetramer and both possible dimers [38]. Recent measurements of self-association constants of mouse arrestin-1 yielded K_d dimer_  = 57.5 µM and K_d tetramer_  = 63.1 µM [34]. These values allow the calculation of the half-life of the dimer and tetramer [48], both of which turn out to be on the order of 12 ms. Thus, arrestin-1 self-association also cannot account for the multi-second times of half-recovery in Tr-4^Arr−/−^ mice (Table 1).

Sub-cellular distribution of arrestin-1 in rods is strictly light dependent. In the dark, arrestin-1 is predominantly located in the inner segment, perinuclear layer, and synaptic terminals, with relatively small fraction, estimated at 2–4% [12], [49], 9% [11], or ∼15% [10], residing in the OS. Prolonged bright illumination triggers the translocation of the majority of arrestin-1 to the OS [20], [49], [50]. Different lines of evidence suggest that arrestin-1 movement is either energy-independent, driven by diffusion [49], may involve active transport [51], or possibly diffusion with active gating in the cilium [11]. Considering that in the light and dark arrestin-1 in the rod is at disequilibrium [52], it is clear that, regardless of the mode of transportation, its distribution must be determined by the interactions with non-mobile partners: otherwise the diffusion would quickly ruin concentration gradients created by any mechanism [53]. Arrestin-1 binds rhodopsin at 1∶1 ratio [54], [55], and the molar amount of arrestin-1 that can translocate to the OS in the light is limited by the amount of rhodopsin present in this compartment [12], indicating that rhodopsin is the immobile binding partner that holds arrestin-1 in the OS in the light. Arrestin-1 binds several proteins present in the cell body, including polymerized tubulin (microtubules) [56], [57], c-Jun N-terminal kinase [58], ubiquitin ligases Mdm2 [58] and parkin [59], calmodulin [60], N-ethylmaleimide-sensitive factor [61], and enolase1 [62]. Among these, however, tubulin appears to be the only sufficiently abundant protein to serve as an “anchor” for arrestin-1 expressed at 0.8∶1 ratio to rhodopsin [10]-[12]. High concentration of arrestin-1 in the compartments particularly rich in microtubules (the inner segment, perinuclear area, and synaptic terminals [63]) in the dark supports this notion. Arrestin-1 translocation is a relatively slow process that takes many minutes [11], [49], [50]. Thus, in dark-adapted animals used in this study arrestin-1 already present in the OS must be responsible for signal shutoff. Microtubules are not abundant in the OS, but several bundles near the outer membrane extend along the full length of the OS and the axoneme [21], [63]. Interestingly, arrestin-1 association with microtubules in the OS was previously reported [64], [65]. The diameter of mouse rod OS is ∼1.4 µm [31], so that arrestin-1 bound to these microtubules would need to diffuse for up to 0.7 µm before reaching rhodopsin. This would take seconds [31], which matches the observed delay of photoresponse recovery in Tr-4^Arr−/−^ mice, as compared to the Tr-12^Arr−/−^ animals (Table 1), fairly well. Importantly, the observed delay of several seconds (Table 1) cannot be explained by arrestin-1 diffusion into the OS from the inner segment: the time arrestin-1 would require to diffuse over the ∼23 µm length of the rod OS would be significantly longer. Even if one takes into account that in Tr-12^Arr−/−^ and Tr-4^Arr−/−^ mice the OS are shorter (∼17 µm and 14 µm in the middle retina, respectively [10]), arrestin-1 diffusion across this distance would take minutes before it can quench rhodopsin in the OS. Although the exact amount of polymerized tubulin in the OS is unknown, if it is present in excess over arrestin-1 at any concentration that significantly exceeds K_D_ of arrestin-1 binding [66], the majority of arrestin-1 would be recruited to microtubules. Thus, the simplest model that accounts for our data is that there are two distinct pools of arrestin-1 in the OS. At least 2.5% is bound to the microtubules at the plasma membrane, whereas the rest is distributed throughout OS cytoplasm, with only the latter being available to quench rhodopsin signaling on the millisecond timescale. In Tr-4^Arr−/−^ animals microtubules take up most of the arrestin-1 present, leaving relatively little immediately available to rhodopsin. This slows down shutoff by the time necessary for arrestin-1 diffusion across the OS. In contrast, in Tr-12^Arr−/−^ mice and higher expressors the microtubules in the OS apparently saturate by the amount of arrestin-1 roughly equal to that present in Tr-4^Arr−/−^ animals, allowing the rest of arrestin-1 to freely distribute in the cytoplasm to be immediately available for rhodopsin shutoff.

In summary, our data suggest the existence of two distinct pools of arrestin-1 in dark-adapted mouse outer segments. To the best of our knowledge, so far only one genetically modified mouse line where rhodopsin shutoff was made rate-limiting was described: mice with low expression of GRK1/2 chimera [67]. In Tr-4^Arr−/−^ mice we made rhodopsin shutoff the rate-limiting stage of photoresponse recovery by low expression of arrestin-1. Collectively, these results strongly support the idea that both phosphorylation and arrestin binding are necessary steps in rhodopsin shutoff.

## Materials and Methods

### Ethics Statement

Animal research was conducted in compliance with the NIH Guide for the Care and Use of Laboratory Animals and approved by the Vanderbilt University Institutional Animal Care and Use Committee/Office of Animal Welfare Assurance (protocol ID M/06/091).

### Generation of transgenic mice expressing arrestin-1 at different levels

The generation of transgenic animals expressing arrestin-1 at various levels under the pRho4-1 rhodopsin promoter has been described previously [10], [12], [49], and the arrestin-1 content in the dark-adapted OS of these mice was quantified by Western blot [10].

### Electroretinography (ERG)

Electroretinograms were recorded from 6 to 8 week old mice reared in 12/12 light-dark cycle (90±10 lux in the cage during light period). Animals were dark-adapted overnight, as described [10], [68]. Mice were anesthetized under dim red light by ip injection of (in µg/g body weight) 15–20 ketamine, 6–8 xylazine, 600–800 urethane in PBS. The pupils were dilated with 1% tropicamide in PBS before and throughout the experiment. An eye electrode made with a coiled 0.2 mm platinum wire was placed on the cornea, a tungsten needle reference electrode was placed in the cheek, and ground needle electrode in the tail [24], [30], [69]. ERG data was collected using the electrophysiologic system UTAS E-3000 (LKC Technologies, Inc.) connected to a Ganzfeld chamber that produced brief (from 20 µs to 1 ms) full field flash stimuli. The various light intensities used were calibrated by the manufacturer and computer controlled. Because mice are sensitive to temperature, animals were placed on a heating pad connected to a temperature control unit to maintain the temperature at 37–38°C throughout the experiment to reduce variability.

The double flash recording was used to analyze the kinetics of recovery [24], [25], [28], [70], [71]. A test flash was delivered to suppress the circulating current of the rod photoreceptors. The recovery was monitored by delivering a second (probe) flash after various time intervals between the two flashes, that ranged from 200 to 120,000 ms. The intensity of the test flash was either −0.8, −0.4, 0, or +0.4 *log*cd*s/m^2^, corresponding to ∼160, ∼400, ∼1000, and ∼2500 photoisomerizations per rod [30]. The following probe flash was 0.65 *log*cd*s/m^2^, corresponding to ∼4,500 isomerizations per rod [30]. Sufficient time for dark adaptation was allowed between trials, as determined by the reproducibility of the response to the test flash. Time-to-peak (implicit time) of the a-wave at the intensity of the probe flash was not significantly different in all four genotypes. This finding along with the shape of the a-wave indicates that the intrusion of b-wave and oscillation potentials [25], [28] did not differentially affect different genotypes. The normalized amplitude of the probe flash a-wave was plotted as a function of time between the two flashes. Instead of fitting the data points to a theoretical equation, which is inevitably based on certain assumptions that may not be correct for all of the genotypes used, we fitted curves with polynomial nonlinear regression using GraphPad Prism (Version 4.0) and considered R^2^>0.95 as a criterion for a good fit. The rate of recovery was characterized by the time interval necessary for half recovery (t_half_), as described [10], [24], [28], [68].

### Statistical Analysis

The data were analyzed by for each light level separately by one-way ANOVA with Genotype as main factor. To examine the change in recovery time with light intensity, the data for each genotype were analyzed separately with Light as main factor. Means were compared using Bonferroni post hoc test with correction for multiple comparisons. In all cases, p<0.05 was considered significant.
